# Experimental Nanopulse Ablation of Multiple Membrane Parasite on Ex Vivo Hydatid Cyst

**DOI:** 10.1155/2018/8497283

**Published:** 2018-02-07

**Authors:** Xinhua Chen, Ruiqing Zhang, Hao Wen

**Affiliations:** ^1^Department of Hepatobiliary and Pancreatic Surgery, Key Laboratory of Combined Multi-Organ Transplantation, Ministry of Public Health, The First Affiliated Hospital, Zhejiang University, Hangzhou, Zhejiang 310003, China; ^2^Hepatobiliary & Hydatid Department, Digestive and Vascular Surgery Centre, Xinjiang Key Laboratory of Echinococcosis, The First Affiliated Hospital of Xinjiang Medical University, Urumqi, Xinjiang 830011, China

## Abstract

The impact of ultrashort nanopulse on cellular membrane is of biological significance and thus has been studied intensively. Different from cell study, this ex vivo study aims to investigate the biological effects of nanosecond pulsed electric field (nsPEF) on an independent multimembrane parasite, human hydatid cyst, to observe the unique influence of nanopulse on macromembrane structure, permeabilization, and biochemistry. The 300 ns nsPEF was delivered on an experimental model of single human hydatid cyst ex vivo with eight different parameters. Then pathological changes during 7 days of 48 parasite cysts were followed up after nsPEF. The laminated layer, the germinal layer, the protoscolex, and cyst fluid were evaluated by the morphological, pathological, and biochemical measurements. The parameter screening found that nsPEF can damage hydatid cyst effectively when the field strength is higher than 14 kV/cm. When nsPEF is higher than 29 kV/cm, nsPEF destroy hydatid cyst completely by collapsing the germinal layer, destructing protoscolices, and exhausting the nutrition.

## 1. Introduction

*Echinococcus granulosus* is a zoonotic disease worldwide. It is highly prevalent in stock raising areas such as Middle East and Australia [1]. It is a neglected tropical disease [2] which can cause health and economic loss [3]. China accounts for a considerable part of the global loss [3]. The recent epidemiological survey showed that* Echinococcus granulosus* occurrence is high in northwest China, becoming a serious public health and economic problem in Xinjiang, China [4], and an effective treatment is urgently needed.

Hydatid disease is caused by the* Echinococcus* tapeworms. The most common type, cystic echinococcosis, is caused by* E. granulosus*. Human are accidentally infected by ingesting parasite eggs from definitive hosts (e.g., dogs and sheep) by oral-fecal route. Parasite eggs travel in human digestive system, hatch in duodenum, grow up as larvae, and pass through intestinal wall into to liver with blood stream. About 75% cases affect liver [1]. It can spread to lung, brain, or bone. In humans, the size of hydatid cysts varied from 2 to 35 cm (1 to 14 inches) depending on the location and space. The rupture of cyst can cause anaphylaxis, cyst infection, and biliary obstruction, which is life-threatening.

Current treatments against hydatid disease include chemotherapy and surgery. It is difficult for the antiparasite medicine to penetrate into the cystic echinococcosis to kill the protoscolices due to multiple layers of cyst [5]. Therefore, the antiparasite medicine can only be used as an optional treatment. Surgery is still the major treatment [6]. The parasite cyst must be opened to aspirate daughter cysts and inject antihelminthic agent. Repeated laparotomy has to be performed to remove the parasite cyst and affected liver segment. As a consequence of open surgery severe complications such as trauma, abscess, biliary system damage, and interoperation contamination occur frequently.

Nowadays the surgery approaches changed gradually from being destructive to constructive. The minimally invasive treatment provides excellent cure rates with minimal morbidity and mortality [7]. PAIR (puncture aspiration injection reaspiration) has been recommended by WHO as an alternative option for hydatid cysts. PAIR with chemotherapy is more effective than open surgery in terms of disease recurrence, morbidity, and mortality [8, 9]. In addition to PAIR, new treatment methodologies are introduced to ablate hydatid cyst, for example, radio frequency [10], microwave [11], cryoablation [12], and HIFU [13–16]. The extreme temperature was used in order to destroy the germinal layer of the parasite but these temperature-based technologies might also cause thermal damage on the neighbor organs or heat sink effect in the blood vessel [17].

Nanosecond pulsed electric field is a novel minimally invasive ablation technology [18]. Quite different from the traditional thermal ablation strategies, nsPEF can ablate the lesion without accumulating much joule heating in the target area. The nonthermal nsPEF ablation has the special advantages in ablating lesions where there are vital blood vessels, bile duct, intestine, and ureter with radiological guidance, making it an ideal candidate for ablating lesions near liver hilum. nsPEF has been mostly used in ablating malignant tumors [19] ant their metastasis [20, 21].

The bioeffects of nsPEF caused on cellular structures, for example, phosphatidylserine externalization, nanopore forming, membrane blebbing, and cell apoptosis, have been intensively studied. So far it was mainly applied in tumor ablation [18–21]. In this groundbreaking study, nsPEF, for the first time, is used to ablate benign parasite lesion, hydatid cyst, which has an active proliferation in the germinal layer.

In this study, 48 single hydatid cysts were chosen as the experimental model to investigate the biological effect of nsPEF on* Echinococcus granulosus*.

Hydatid cyst can survive in extreme external conditions even it is removed from the host. It grows very slowly in years and will not significantly change by itself in short time, for example, days. The single cyst is a close system including (1) acellular connective tissue, (2) germinal layers, (3) viable protoscolices, and (4) metabolic cyst fluid, making itself an ideal independent biological system. Whether and how nsPEF affect hydatid cyst viability and cyst permeability are unknown. Our hypothesis is that hydatid cyst is a good ex vivo multimembrane model to demonstrate the visible morphological and biofunctional changes caused by nsPEF compared with tiny cellular model or suppositional simulation. This initial investigation ex vivo traced cyst morphological changes and fluid biochemistry changes after nsPEF treatment.

In this study, 48 single hydatid cysts were chosen as the experimental model to investigate the biological effect of nsPEF on* Echinococcus granulosus*. Hydatid cyst is of a semipermeable structure that allows the nutrition ingredients to pass from the host to the parasite but it can prevent macromolecules from entering the cyst.

In this experimental study, the eight different nsPEF treatment parameters were screened. The 300 ns nsPEF was delivered at frequency 1 Hz with different field strengths and different pulse numbers. The effect of nsPEF on laminated layer, the germinal layer, the protoscolex, and cyst fluid were observed. After nsPEF treatment, the morphology, pathology, and biochemical changes were followed up for 7 days to evaluate the efficacy after nsPEF ablation. This study tried to illuminate the possible mechanism and provide the feasible treatment strategy for the further preclinical studies.

## 2. Experimental

### 2.1. Ethical Statement

The experimental protocol was approved by the Ethical Committee of the First Affiliated Hospital of Xinjiang Medical University (approved project number: 20141217003). The experiments have been conducted according to the principles expressed in the Declaration of Helsinki.

### 2.2. Experiment Design

**Figure 1:**
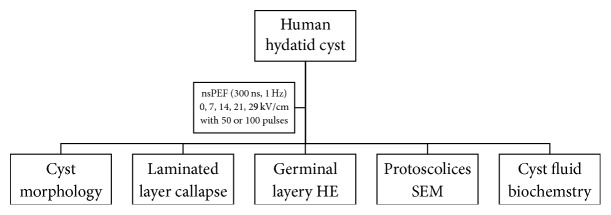
The flowchart of the experiment design.

The experiment design was illustrated in Figure 1. The nsPEF was produced by a Blumlein line generator. The lab equipment design and typical pulse shape were described previously [22]. The 300 ns nsPEF was delivered to the hydatid cyst with a pair of electrodes under different nsPEF electric field strengths (0, 14, 21, and 29 kV/cm) and pulse numbers (50 and 100 pulses). After nsPEF treatment, the cysts were maintained in the incubator for continuous morphological observation under light microscope. After 7 days the cysts were cut open to get the four different parasite components (laminated layer, germinal layer, protoscolices, and cyst fluid) for further scanning electron microscopy (SEM) and pathological and biochemical analysis.

### 2.3. Collection of* Echinococcus granulosus* Cysts

The parasite cysts were collected from naturally infected human hydatid cysts during an open surgery in sterile operation room at the First Affiliated Hospital of Xinjiang Medical University. The sample collection written informed consent forms and picture release agreement had been signed in advance. The single cysts were separated. The 48 single cysts of 1 cm with thin wall, clear fluid, and transparent capsule were chosen for nsPEF treatment.

### 2.4. The Maintenance of* Echinococcus granulosus* Cyst Ex Vivo

The hydatid cysts were cultured in the 6-well culture plate. Total 5 mL RPMI1640 medium containing 10% fetal bovine serum was added. The plate was kept in 37°C 5% CO2 incubator. After nsPEF treatment, the cyst morphological changes were checked every day under the converted light microscope.

### 2.5. The nsPEF Treatment Ex Vivo

**Figure 2:**
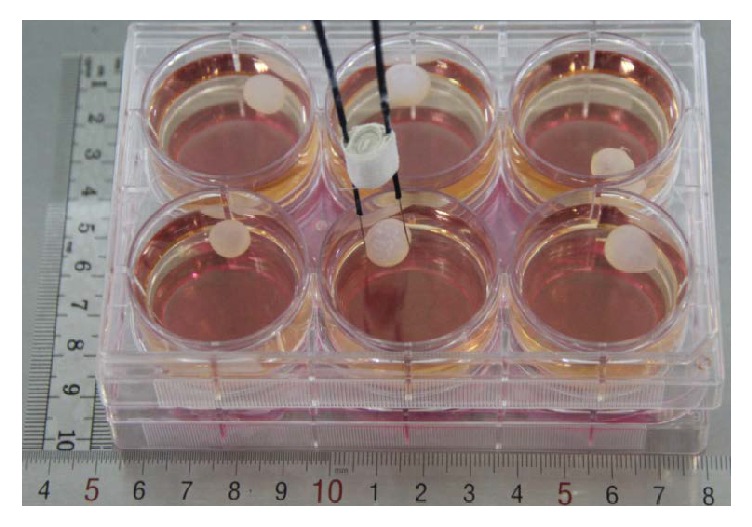
*The nsPEF treatment on the hydatid cyst ex vivo*. The hydatid cysts of good elasticity with diameter around 10 mm, bright and transparent, were placed in 6-well culture plate for nsPEF treatment.

The pulse generator was made in Leibniz Institute for Plasma Science, Germany, based on the same Blumlein design and treatment parameters described previously [22]. The pair electrodes were applied on a 1 cm sized cyst (Figure 2). The previously optimized treatment parameters were as follows: 300 ns, square pulse, and 40 kV/cm [22].

The single hepatic hydatid cyst was removed completely and put into the culture plate and treated immediately by nsPEF ex vivo. The nsPEF generator and electrodes were made by Xinjiang Nanosecond Pulsed Power Institute.

The 300 ns nsPEF was delivered 1 Hz with four different nsPEF electric field strengths (0, 14, 21, and 29 kV/cm) and two pulse numbers (50 and 100 pulses), as shown in Table 1. The sterile electrodes were placed into the culture medium with the single cyst in between the two electrode probes (Figure 2).

### 2.6. Cyst Collapse Observation and Collapse Rate Calculation

After the nsPEF treatment, the 48 cysts were observed under the microscope (OlympusBX51, Tokyo, Japan) and the result was recorded by a camera (LeicaDC350F, Wetzlar, Germany). Images were quantitatively analyzed according to the collapse rate. The collapse rate is (the number of cysts with collapse on the cyst wall/the total cysts treated by nsPEF × 100%).

### 2.7. The Pathological Changes Evaluation by H&E and Scanning Electron Microscopy (SEM)

On the 7th day, the specimens were fixed with paraformaldehyde and glutaraldehyde, respectively. The ultrastructural changes were observed by H&E stain and SEM, respectively. Briefly, for ultrastructure observation by scanning electron microscope, the separated protoscolices were fixed in 2.5% glutaraldehyde 4°C for overnight and then put in 2% OsO4 for 24 hours, followed by dehydration in series of ethanol. After coating with gold, pictures of the samples were taken on a scanning electron microscope (JSM6380, Japan) at the Electron Microscope Center, Xinjiang Medical University. For H&E stain, the separated hydatid cysts were fixed in 10% formalin and then embedded in paraffin, cut into 5 *μ*m slide sections, and stained with hematoxylin and eosin, and pictures of them were taken under light microscopy to evaluate tissue structure and pathological changes.

### 2.8. The Fluid Cyst Biochemistry Analysis

Before nsPEF treatment and on the 7th day after nsPEF treatment, the cysts were cut open and the cyst fluid was aspirated for the biochemistry analysis. The basic nutrition markers for parasite survival (protein, glucose, and pH) were tested by automatic biochemical analyzer AU2700, (Olympus, Tokyo, Japan). The instrument was calibrated by the quality-control liquid before testing the hydatid cyst fluid. The biochemical results in nsPEF-treated group (under the previously proved effective parameter as 29 kV/cm and 100 pulses) were compared with the control group (cysts without any nsPEF treatment but maintained for the same time period). The self-parallel blank control was also set up to deduct the effect of culture time and background noise.

### 2.9. Statistical Analysis

All data are present mean ± SEM from 3 independent experiments. Data were analyzed with SPSS 17.0 software. The comparison was considered statistically different when *P* < 0.05 by Student's *t* test.

## 3. Results

### 3.1. The Morphological Changes in the First Week

**Figure 3:**
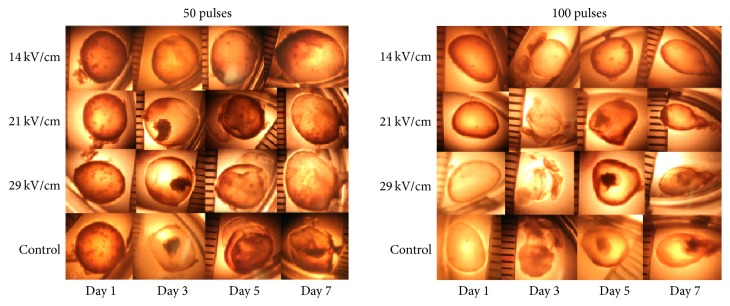
*Hydatid cyst morphological changes in the first week under different treatment parameters*. All together 48 single different hydatid cysts were included in the morphological study. The living parasite is characterized with smooth cyst wall to keep normal metabolic function. The dead parasite is characterized with the cyst wall distortion and dysfunction. The morphological change is the direct diagnosis. When functional and alive, the parasite cyst is smooth. When dead and dysfunctional, the cyst wall is curved and distorted.

The cyst wall has multiple layers and provides the physical defense for parasites. A single hydatid cyst was observed under light microscope. The outermost layer is a thin connective tissue of host origin, pericyst; the middle layer, laminated membrane; and the inner layer, germinal layer were hard to be differentiated, but the hydatid cyst contents such as cyst fluid, protoscolices, and daughter cysts were visible (Figure 3 control group). After nsPEF, cyst homogeneousness was disrupted (14 kV, 50 pulses, day 3, day 5, and day 7). The cyst fluid was colorless and transparent in control group but cloudy in nsPEF-treated group due to the detachment of the daughter cyst and protoscolices (21 kV/cm 50 pulses, day 3 and day 7). When the nsPEF strength and pulse number increased, the inner contents became condensed (14 kV/cm, 100 pulses, day 5 and day 7; 21 kV/cm, 100 pulses, day 3, day 5, and day 7); the cyst wall was fragmented and cyst ruptured (Figure 3, 29 kV/cm, 100 pulses, day 3, day 5, and day 7).

### 3.2. The nsPEF Collapsed the Cyst Wall

To quantitatively describe the result in Figure 3, the collapsed rate was calculated. The collapse rate is (the number of cysts with collapse on the cyst wall/the total cysts treated by nsPEF × 100%). In this study, collapse rates of the cystic wall were the highest in nsPEF-treated groups (29 kV/cm 100 pulses) on days 1, 3, 5, and 7, compared with control groups on the same day. There was significant difference (*P* < 0.05), indicating the dose effect of nsPEF. The nsPEF at 29 kV/cm and 100 pulses was the most effective parameter which was then used for the further experiment.

Normal hydatid cysts are fluid-filled and with a uniformly thin and smooth wall to round shape. To better clarify the nsPEF caused bioeffects, we derived human hydatid cysts, kept ex vivo, and analyzed the morphological changes taking place in response to the exposure to 8 different nsPEF treatment parameters. We identified 48 living hydatid cysts freshly removed from hydatid patients, each with a single parasite cyst of 1 cm in diameter. During 7-day follow-up period, all nsPEF-treated cysts were histologically evaluated. The cyst wall morphological changes were visualized by microscopy and evaluated by image analysis. nsPEF induced morphological changes indicating damage of multiple membranes. These findings indicate that nsPEF exerts time-dependent changes in the cyst wall, which may contribute to parasite dysfunction and death.

### 3.3. The nsPEF Treatment Collapsed the Laminated Layer and Germinal Layer

**Figure 4:**
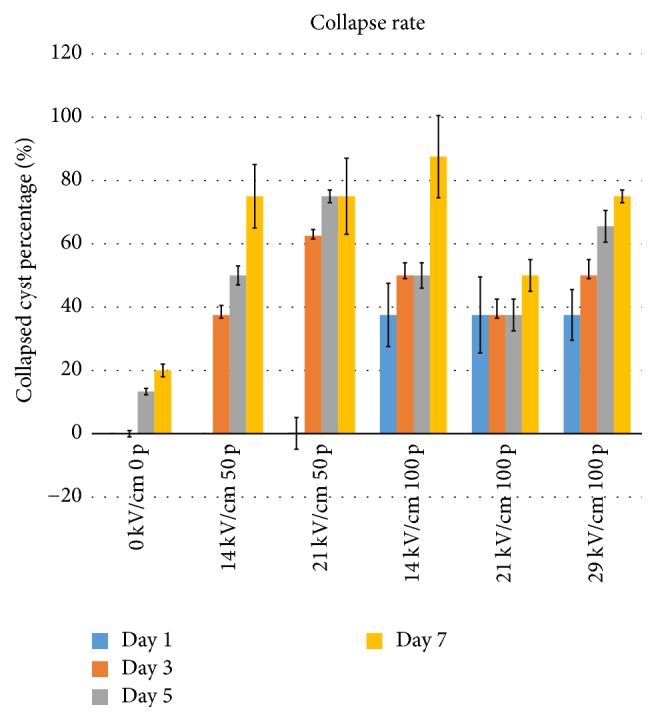
*The quantitative analysis of hydatid cyst wall collapse*. The statistical data was made by the number of cysts with distorted wall (dysfunctional) versus cysts with smooth cyst wall. The quantitative data were also summarized. All data are present mean ± SEM from 3 repeated measurements.

**Figure 5:**
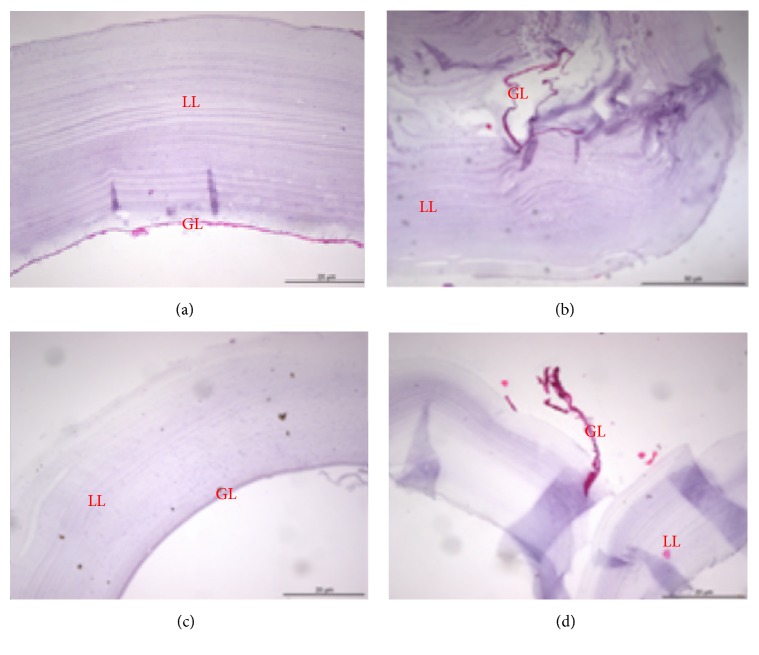
*H&E stain of laminated and germinal layers after nsPEF treatment*. LL, laminated layer; GL, germinal layers. (a) The control group on the 7th day. (b) The nsPEF-treated group (14 kV/cm, 50 pulses) on the 7th day. (c) The control group on the 7th day. (d) The nsPEF-treated group (29 kV/cm, 100 pulses) on the 7th day.

H&E stain confirmed that nsPEF treatment disrupted the continuity of cyst wall which was made of laminated layer and germinal layer (Figure 4 and Figure 5). As shown in Figure 5, the germinal layer was shed from the stratum corneum, and the cyst structure was blurred, while, in the control group, the cyst structure and outline were clear. The mild nsPEF treatment (14 kV/cm, 50 pulses) caused the partial collapse in which the germinal layer was twisted but not fragmented (Figure 5(b)). With the higher nsPEF (29 kV/cm, 100 pulses) the germinal layer was ruptured (Figure 5(d)).

### 3.4. The nsPEF Treatment Destroyed the Protoscolices

**Figure 6:**
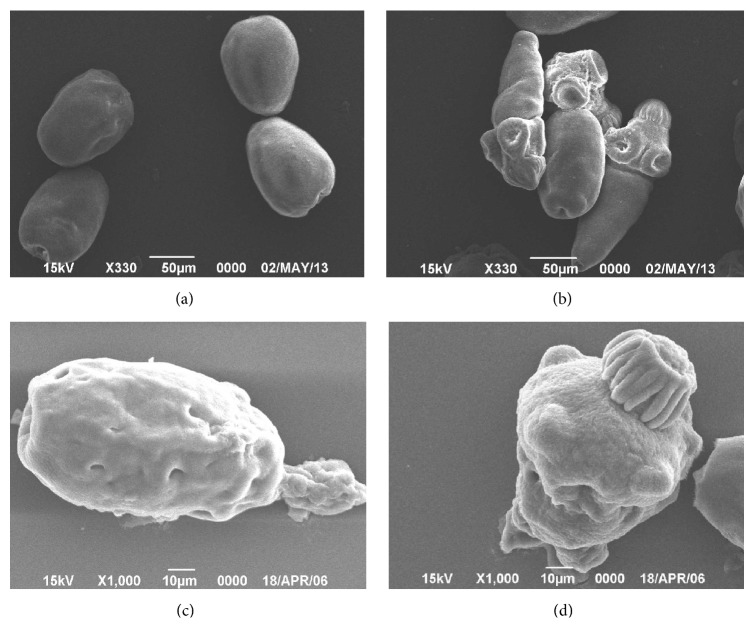
The destruction of protoscolices by scanning electron microscopy. (a) Normal protoscolex without nsPEF (retraction type). (b) Normal protoscolex without nsPEF (eversion type). (c) nsPEF-destroyed protoscolex (retraction type). (d) nsPEF-destroyed protoscolex (eversion type).

SEM confirmed that nsPEF treatment destroyed protoscolices. As shown in Figure 6, without nsPEF, the normal protoscolex has two different appearances: retraction type with sucker hook inside (Figure 6(a)) and eversion type with sucker hook outside (Figure 6(b)). Even the mild nsPEF (14 kV/cm, 50 pulses) can destruct them (destroyed retraction type in Figure 6(c) and destroyed eversion type in Figure 6(d)), as shown in Figures 6(c) and 6(d). The previously retracted sucker hook was fragmented and flew out. The parenchymal tissue in the body was punctured. As shown in Figure 6(d), the protoscolex has bulges and sucker detachment.

All together 48 single different hydatid cysts were included in the morphological study, SEM is too time- and labor-consuming to get statistical analysis. Only the protoscolex with the most intensive nsPEF treatment (#8 parameter in Table 1) was shown in Figure 6.

### 3.5. The nsPEF Exhaust Basic Nutrition in the Cyst Fluid

**Figure 7:**
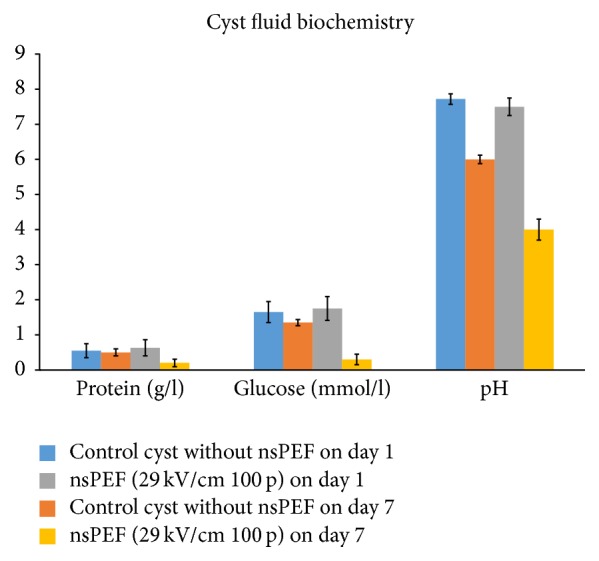
*The protein, glucose, and pH decreased in the nsPEF-treated groups*. The protein, glucose, and pH in the cyst fluid were measured by an automatic biochemical analyzer OlympusAU2900. All data are present mean ± SEM from 3 repeated measurements.

Because cyst fluid makes up the inner environment for parasite and exchanges nutrition with the host, an understanding of how the essential components of cyst fluid change after nsPEF treatment will help to predict the parasite survival. Three basic biochemical, markers, protein, glucose, pH, are the basic nutritional elements. They can reflect the parasite metabolism. After nsPEF treatment they were significantly lower than those in control group (Figure 7). To deduct the confounding factor of culture in the medium ex vivo, the self-parallel controls cultured at the same period of time with the same culture condition were also set up. pH decreased significantly over time in control group (*P* < 0.05) while, after nsPEF treatment, the pH drop became even more sharply (*P* < 0.001). Notably, for each nutrition component, the serum equivalent from human host was also tested. The protein in the hydatid cyst fluid was lower than 1% of that in the human host serum (parasite 0.5 g/L versus host serum protein 48 g/L), glucose 20% (parasite 1-2 mmol/l versus host serum 6.3 mmol/L). The pH values were similar (parasite 7.35 vesrus host 7.45), indicating that, before nsPEF treatment, parasite has set up its own relatively independent metabolism environment inside the cyst to keep the long-term symbiotic relationship with human host. After nsPEF, the nutrition production cycle and metabolism balance in the inner environment were broken.

## 4. Discussion

A detailed understanding of dose effect of nsPEF on hydatid cyst facilitates development of treatment strategies for hydatid disease. The hydatid cyst is also a good close system to study the biological effect of nsPEF. So this study is of medicine and mechanism importance.

In general, when patients have the higher parasite load, the more severe hydatid disease symptoms will occur in human. The host defense fights to reduce the parasite load to lower levels. Human patients generally fail to eliminate the protoscolices completely. The germinal layer makes the parasite production continue. In this study nsPEF targets two of the parasite defenses by (1) destructing the hydatid cyst wall so that the parasite's protective fence is broken and (2) destroying the protoscolex so that the germinal sources are damaged.

In accordance with germinal activity, World Health Organization classified the cystic echinococcosis as CE1–CE5 with their specific ultrasound image characters [23]: CE1, a single cyst with a constant cyst content density; CE2, wheel sign; CE3, floating lotus sign; CE4, heterogeneous without daughter cysts; CE5, calcification of the wall. Among all the 5 types, the CE1 type is early active stage in which the germinal function is the most active. The active protoscolices inside the cyst are the key factor that causes cyst growth, expansion, rupture, contamination, and recurrence. So in this study CE1 type cyst was collected from human as the experimental model.

The benzimidazoles such as mebendazole and albendazole have been developed as antihydatid disease drugs recommended by WHO [24]. Some new derivatives of benzimidazoles and liposomal drugs and Chinese herbs were also introduced into experimental studies. But their side effects such as jaundice, fever, dizziness, hair loss, elevated transaminases, proteinuria, teratogenic, abdominal pain, diarrhea, nausea, vomiting, and leukemia hindered the long-term use. Moreover, albendazole showed poor intestinal absorption and low drug concentration in blood and liver. The follow-up studied indicate that only 30% of patients were cured by albendazole, 30–50% had improvement, and 20–40% had no change at all [25]. Therefore, although drug treatment has achieved a certain effect, the clinical efficacy is still not satisfactory. Destructing the hydatid cyst wall is another focus of antihydatid disease treatment. The destroyed cyst wall can make the protoscolices lose the protection fence, making the chemotherapeutic agent penetrate into the cyst. But the damage of the cyst wall must be accompanied with the killing of protoscolices because the active protoscolices may spread and plant in abdominal cavity.

Previously high-energy ultrasound focused knife, radio frequency ablation, and cryoablation techniques have been tried in hepatic hydatid cysts [12–16]. These thermal methodologies also caused biliary tract, blood vessel, and intestine injury [17] in adjacent organs.

Nanosecond pulse ablation is a novel method of ablation technique that is independent of heat accumulation [25]. It applied electrical field in ultrashort pulses so that the biological cells with their own electric activity react with nsPEF based on the cell membrane structure. nsPEF causes the different charges on inner and outer cell membrane so that the cell membrane structure is distorted and changed. Depending on the pulse duration, electric field strength, and pulse number, nsPEF interacts with cell membrane and presents a variety of intracellular biological effects. Most interestingly, the cellular and plasma membrane permeability changes in different circumstances.

*Echinococcus granulosus* eggs were spread into environment from infected dog feces. The eggs can stay alive in soil, water, and vegetable and survive snow and freezing conditions. The eggs can survive at least a year in the environment as they are highly resistant to environmental stress. Egg survival time is increased in damp conditions (e.g., near watering holes). In this experiment parasite cyst was kept in an ex vivo cell culture system with suitable temperature liquid and nutrition which ensure the viability.

Our hypothesis is nsPEF can disrupt the hydatid cyst wall and change the multiple cyst layers, leading to the killing effect on protoscolices in the germinal layer and facilitate the membrane permeability for drug penetrating into the cyst.

First, this study set up an ex vivo model for nsPEF biological effect investigation. Due to the complexity of the hydatid cyst, it is hard to set up theoretical explanation by mathematical models without the knowledge of the pathological structure. The hydatid cyst model elucidates its efficacy on ablating hydatid cysts with quantitative experimental parameters. Another independent* in vivo* research [26] confirmed the feasibility of applying nsPEF treating parasite disease in liver.

Second this study tried different treatment parameters. The pulse duration was fixed as 300 ns; the frequency was fixed as 1 Hz. The field strengths were changed (0 kV/cm, 7 kV/cm, 14 kV/cm, 21 kV/cm, and 29 kV/cm) as well as the pulse number (50 and 100 pulses). The data showed a dose effect. The higher electric field and more pulse numbers lead to more significant killing parasite effect. 29 kV/cm and 100 pulses were the effective treatment. The nsPEF showed a killing effect on the protoscolices when the field strength was higher than 14 kV/cm. The killing rate increased in accordance with the increase of the voltage. The morphological change of the protoscolices was significant. When the electric field intensity increased up to 29 kV/cm, the protoscolices were totally destroyed. The stratum corneum lamellar structure of the parasite cyst was destroyed. The stratum and outer layer were broken accordingly. Several typical characteristics of nsPEF, such as voltage amplitude and the number of pulses, will affect the effect of ablation therapy. This ex vivo study provides the preliminary dose reference for further clinical study.

Third, this study proved that nsPEF has the following advantages: nsPEF treatment did not burn the hydatid cyst; the collagen fibers and other connective tissue components in the hydatid cyst were not denatured.

The biological effects of nanosecond pulse electric field have been intensively investigated [18, 27–30]. The key results of this study showed that the nsPEF can also affect parasite. In terms of mechanism, nsPEF targets at least four parasite components including both outer defense wall and inner germinal source. If the protoscolices are not killed the hydatid cyst will keep on growing and spreading. The germinal layer can continue to produce the protoscolices and germinal sac. They either attach to the cyst wall or fall to suspend in the cyst fluid. In this study, nsPEF cause the destructive effect on protoscolices and they lose their viability after nsPEF. After nsPEF, the continuity of the cyst wall was destroyed and the germinal layer was shed from the stratum corneum. The cyst wall structure was blurred, indicating that nsPEF collapsed the multiple-layer cyst wall. Protein, glucose, and pH are the most basic markers for evaluating any inner environment such as plasma for cell, blood for human, and cyst fluid for hydatid larva. They contribute to maintain the pressure, keep the balance, and provide nutrition for the larvae survival. nsPEF damaged the cyst wall, collapsed the germinal layer, and destroyed the protoscolices, as a result, the nutrition in the cyst fluid was exhausted, contributing to the vicious circle of more weakened metabolism. nsPEF may have mechanical, electrical, and immunological effect on hydatid cyst, but further animal studies* in vivo* are needed to determine its contribution in parasite eradication.

## Figures and Tables

**Table 1:** The summary of eight different nsPEF treatments on 48 cysts.

Treatment parameter	Pulse duration (ns)	Frequency (Hz)	Electric field strength (kV/cm)	Pulse number	Different cyst treated by nsPEF
#1	300	1	0	50	6
#2	300	1	14	50	6
#3	300	1	21	50	6
#4	300	1	29	50	6
#5	300	1	0	100	6
#6	300	1	14	100	6
#7	300	1	21	100	6
#8	300	1	29	100	6
